# Curcumin and Cognitive Function: A Systematic Review of the Effects of Curcumin on Adults With and Without Neurocognitive Disorders

**DOI:** 10.7759/cureus.67706

**Published:** 2024-08-25

**Authors:** Aida J Francis, Chithra Sreenivasan, Aneri Parikh, Osamah AlQassab, Tatchaya Kanthajan, Manorama Pandey, Marcellina Nwosu

**Affiliations:** 1 Internal Medicine, California Institute of Behavioral Neurosciences and Psychology, Fairfield, USA; 2 Clinical Research, California Institute of Behavioral Neurosciences and Psychology, Fairfield, USA

**Keywords:** curcumin, serum biomarkers, cognitive health, dementia, neurocognitive disorders

## Abstract

This systematic review investigates the effect of curcumin on neurocognitive exams and inflammatory serum biomarkers in adults 18 years and older. We search PubMed, Science Direct, Google Scholar, Cochrane Library, and Multidisciplinary Digital Publishing Institute. Modeling the Preferred Reporting Items for Systematic Reviews and Meta-Analyses guidelines (PRISMA), we screened 1,284 studies with the keywords "neurocognitive disorders," "dementia," "cognitive health," "serum biomarkers," and "curcumin." We use the revised Cochrane Risk-of-Bias tool (RoB2) and the Newcastle-Ottawa Scale to select 12 open-access full-text articles published within 20 years. We include clinical trials, randomized controlled trials (RCTs), cohort studies, and human studies, excluding nonhumans, other design types, and schizophrenia. Despite gastrointestinal side effects, studies found curcumin significantly improves working memory in the following adult groups: non-demented, metabolically impaired, cognitively impaired, mood impaired, and chemotherapy impaired. Study limitations include variable population characteristics and few trials employing intention-to-treat analysis, emphasizing the need for shared clinical decision-making before curcumin therapy.

## Introduction and background

In 2023, Alzheimer's disease climbed to the fifth most common cause of death among American adults aged sixty-five years and older; in 2024, it maintains fifth rank, with approximately 6.9 million older adults currently living with this disease [1]. Neurocognitive disorders like Alzheimer's disease stem from various medical conditions, and their progression can be measured using cognitive performance evaluations such as the Mini-Mental State Exam (MMSE) and Montreal Cognitive Assessment (MoCA) [2,3]. The molecular pathophysiology of neurocognitive decline involves inflammation and cellular breakdown of nervous tissue, leading to the accumulation of inflammatory markers like beta-amyloid in the blood, which are measured to assess for neurodegeneration. Functional decline can lie dormant for years and affect previously healthy adults at different progression rates, but the cellular damage cannot revert once it ensues [4]. Because there is no standard cure for dementia after the onset of cognitive decline, it is essential to explore preventive treatment options for healthy adults as well as adults with a variety of cognitive disorders [5].

Current treatments for cognitive decline, such as cholinergic agonists like Aricept, temporarily stimulate the brain but do not cure long-term functional deterioration and can cause side effects like bradycardia [5]. Thus, there is a need for less toxic, more affordable, and naturally derived treatments to promote cognitive health. Curcumin, a natural polyphenol found in turmeric, has antioxidant and anti-inflammatory properties, which makes it a promising candidate for preventing and treating cognitive disorders. However, the limited bioavailability of curcumin poses a challenge, easily mitigated with enhancers such as piperine, found in black pepper, and lipid formulations [6,7].

A 2021 systematic review by Tsai et al. found that curcumin has greater benefits with working memory and cognitive speed, but no significant benefits in other cognitive domains. The limited amount of data calls for more clinical trials with an intention-to-treat protocol. Despite these findings, curcumin is not yet widely accepted as a standard treatment for cognitive disorders due to limitations in current clinical trials and minor gastrointestinal side effects. More research is needed to explore the best bioavailable form and effective dose of curcumin across various neurocognitive disorders [8].

With the increase in Alzheimer's morbidity after the coronavirus disease (COVID) pandemic, it is crucial to re-explore the therapeutic benefits of curcumin [1]. This systematic review aims to update and expand on the findings of Tsai et al. by incorporating more recent studies [8]. We include adults aged 18 and older to evaluate the effects of curcumin on cognitive performance and serum biomarkers in diverse patient populations, including those with and without neurocognitive disorders.

## Review

Methodology

This systematic review follows the rigorous Preferred Reporting Items for Systematic Review and Meta-Analyses (PRISMA) guidelines [9].

Selection Criteria

The studies in this review meet the stringent Population, Intervention, Comparator, Outcome, and Study Design (PICOS) criteria, understood in systematic reviews, to ensure a clear and structured approach to study selection and analysis. The population criteria (P) include adults aged 18 years and older, both with and without neurocognitive disorders. The intervention criteria (I) include supplementation with curcumin. The comparator criteria (C) include placebo or alternate supplementation. The outcome criteria (O) include cognitive status measured by cognitive performance exams or inflammatory serum biomarkers. The study design criteria (S) include open-access full-text articles, clinical trials, randomized controlled trials (RCTs), cohort studies, human studies, and studies published in the last twenty years. Given the lack of a cure for long-term cognitive decline, it is crucial to include populations at risk of dementia prior to onset [5]. Thus, we include adults aged 18 years and older, covering various patient populations, including healthy patients and those at risk of or already diagnosed with Alzheimer's disease, as modeled by Tsai et al. [8]. We manually screen relevant articles for inclusion based on the PICOS criteria described.

We exclude interventions other than curcumin, nonhuman models, case reports, case series, meta-analyses, reviews, systematic reviews, studies lacking data on cognitive performance or serum biomarkers, non-open access articles, and non-full-text articles. These exclusions focus on curcumin intervention in neurocognitive disorders and ensure the reliability and relevance of the included studies. Findings show sufficient data from the eleven clinical trials and one cohort study, while other excluded study design types did not enhance or add new information to this review. We also exclude studies involving patients with schizophrenia due to the ongoing fluctuations in mental status that could skew the results of our study in unmeasurable ways, not addressing neurocognitive decline.

Search Strategy

To ensure a comprehensive review, investigators Aida Jeanette Francis (AJF) and Aneri Parikh (AP) independently conducted thorough searches across multiple databases to gather relevant articles for investigating the outcomes of curcumin intervention in neurocognitive disorders. These databases include PubMed, Science Direct, Google Scholar, Cochrane Library, and Multidisciplinary Digital Publishing Institute (MDPI). We searched databases from May 1, 2024, through June 30, 2024, using advanced search techniques and keywords combined with Boolean terms to identify relevant articles. We selected the following keywords for all databases searched: neurocognitive disorders, dementia, cognitive health, serum biomarkers, and curcumin. We used several Medical Subject Heading (MeSH) terms for the PubMed Database to perform an advanced search. Upon further investigation, no MeSH term was available for the keyword cognitive health in the PubMed database. Furthermore, the following medical subject headings in the PubMed database were applied only to the keyword curcumin: "administration and dosage," "adverse effects," "analogs and derivatives," "analysis," "isolation and purification," "metabolism," "pharmacokinetics," "pharmacology," "therapeutic use," and "toxicity." After performing an advanced search, we applied the following selection criteria in the search filters on PubMed: "abstract," "free full text," "clinical trial," "humans," and "from 2004/1/1 - 2025/1/1." For the Science Direct database, we applied the following selection criteria to the search filters: "2004-2024," "other article type," and "open access & open archive." For Google Scholar, we only applied the date restrictions "2004-2024." For Cochrane Library, we applied the following selection criteria to the advanced search limits: "publication year from 2004 to 2024," "Cochrane Library publication date from January 2004 to July 2024", and "trials." We did not use filters or search term modifications for the MDPI database. Table 1 details the search strategy for each database.

**Table 1:** Search strategy MDPI: Multidisciplinary Digital Publishing Institute; MeSH: Medical Subject Headings; ffrft: PubMed "free full text" filter; fha: PubMed "abstract" filter

Database	Date searched	Keywords	Search terms	Filters	Number of studies
PubMed Mesh	6/30/24	Neurocognitive disorders or Dementia or Cognitive health or Serum biomarkers and Curcumin	(("Neurocognitive Disorders"[MeSH Major Topic] OR "Dementia"[MeSH Major Topic] OR "Biomarkers"[MeSH Major Topic]) AND ("curcumin/administration and dosage"[MeSH Terms] OR "curcumin/adverse effects"[MeSH Terms] OR "curcumin/analogs and derivatives"[MeSH Terms] OR "curcumin/analysis"[MeSH Terms] OR "curcumin/isolation and purification"[MeSH Terms] OR "curcumin/metabolism"[MeSH Terms] OR "curcumin/pharmacokinetics"[MeSH Terms] OR "curcumin/pharmacology"[MeSH Terms] OR "curcumin/therapeutic use"[MeSH Terms] OR "curcumin/toxicity"[MeSH Terms])) AND ((ffrft[Filter]) AND (fha[Filter]) AND (clinicaltrial[Filter]) AND (humans[Filter]) AND (2004/1/1:2025/1/1[pdat]))	Abstract, free full text, clinical trial, humans, and from 2004/1/1 - 2025/1/1	2
Science Direct	6/30/24	Neurocognitive disorders or Dementia or Cognitive health or Serum biomarkers and Curcumin	(Neurocognitive disorders or Dementia or Cognitive health or Serum biomarkers) and Curcumin	2004-2024, another article type, and open access & open archive	2
Google Scholar	6/30/24	Neurocognitive disorders or Dementia or Cognitive health or Serum biomarkers and Curcumin	(Neurocognitive disorders or Dementia or Cognitive health or Serum biomarkers) and Curcumin	2004-2024	1180
Cochrane Library	6/30/24	Neurocognitive disorders or Dementia or Cognitive health or Serum biomarkers and Curcumin	(Neurocognitive disorders or Dementia or Cognitive health or Serum biomarkers) and Curcumin	Publication Year from 2004 to 2024, Cochrane Library publication date from January 2004 to July 2024, and trials	100
MDPI	6/30/24	Neurocognitive disorders or Dementia or Cognitive health or Serum biomarkers and Curcumin	(Neurocognitive disorders or Dementia or Cognitive health or Serum biomarkers) and Curcumin	N/A	0

Study Selection Process

Two authors, AJF and AP, comprehensively evaluated each study's internal validity using two quality assessment tools. This review covers two specific study designs: RCTs and cohort studies. The revised Cochrane risk-of-bias (RoB2) tool was applied for randomized control trials, as seen in Table 2 [10]. The Newcastle-Ottawa tool was used for cohort studies, as seen in Table 3 [11].

**Table 2:** RoB2 tool for randomized trials Each RCT was evaluated for bias, receiving a rating of “Low,” “High,” or “Some Concerns” [10]. RoB2: revised Cochrane risk-of-bias; RCT: randomized controlled trial

Selected study	Bias arising from the randomization process [10]	Bias resulting from diverging from the intended intervention [10]	Bias due to missing outcome data [10]	Bias in the approach to measure the outcome [10]	Bias in the selection of the report [10]
DiSilvestro et al. (2012) [12]	Low	Low	Low	Low	Low
Cox et al. (2020) [13]	Low	Low	Low	Low	Low
Rainey-Smith et al. (2016) [14]	Low	Some concern	Some concern	Low	Low
Small et al. (2018) [15]	Low	Low	Low	Low	Low
Kuszewski et al. (2020) [16]	Some concern	Some concern	Low	Low	Low
Lee et al. (2014) [17]	Low	Low	Some concern	Some concern	Low
Thota et al. (2020) [18]	Low	Low	Low	Low	Low
Das et al. (2023) [19]	Low	Low	Low	Low	Low
Ringman et al. (2012) [20]	Low	Some concern	Some concern	Some concern	Low
Alimadadi et al. (2022) [21]	Low	Low	Low	Low	Low
Putri Laksmidewi et al. (2024) [22]	Low	Low	Some concern	Low	Low

**Table 3:** Newcastle-Ottawa quality assessment table for screening cohort studies *Star point rating, a star is awarded for fulfilling each question within each subsection of the Newcastle-Ottawa assessment.

Selected study	Study design [11]	Selection [11]	Comparability [11]	Outcome [11]	Total stars
Ng et al. (2022) [23]	Retrospective cohort	***	*	**	6/9=fair

Data Collection Process

We observed the effect of curcumin on cognitive performance and serum biomarkers in adults 18 and older from 11 RCTs and one retrospective cohort study [12-23]. Two authors, AJF and Osamah AlQassab (OA), independently collected data to include study characteristics, results, and outcomes for each of the 12 selected studies. Table 4 displays each study's characteristics.

**Table 4:** Study characteristics Serial Threes and Sevens and the virtual Morris Water Maze are used for cognitive performance exams; the Profile of Mood States is used for mood assessment. Biocurcuma, Theracurmin, CurQfen®️, and Curcumin C3 Complex are all synthetic copyrighted brands of curcumin. A positron emission tomography radiotracer is used for tracking neurofibrillary tangles and amyloid aggregates in the brain [15]. Cohen's d effect size measures the difference between group means. sICAM: soluble intercellular adhesion molecule; c-Reactive Protein: carbohydrate capsule of the pneumococcus capsule reactive protein; SD: standard deviation; MoCA: Montreal Cognitive Assessment; P: probability value; FDDNP: 2-(1-{6-[(2-[fluorine-18]fluoroethyl)(methyl)amino]-2-naphthyl}-ethylidene)malononitrile; PET: positron emission tomography; BMI: body mass index; DHA: docosahexaenoic acid; EPA: eicosapentaenoic acid; CVR: cerebrovascular reactivity; WM: working memory; R: correlation coefficient; GSK-3β: glycogen synthase kinase-3β; IAPP: islet amyloid polypeptide; HOMA2-IR: homeostasis model assessment of insulin resistance; CGM: CurQfen®️-curcumin; USC: unformulated standard curcumin; MMSE: Mini-Mental State Exam; GI: gastrointestinal; η²: ratio of variance; CICI: chemotherapy-induced cognitive impairment; ∆ median: statistical variability from median; Z: z-score or statistical variability from the mean

Author and year of publication	Population characteristics	Intervention studied	Number of patients	Study design	Results	Conclusion
Disilvestro et al. (2012) [12]	Healthy middle-aged adults (men and “post menopause women”) 40-60 years old	Lipidated curcumin extract (80 mg/day)	38 (19 curcumin and 19 placebo)	four-week randomized and placebo-controlled trial	⁠Curcumin had statistically significant results in plasma and salivary levels as follows: lower beta-amyloid protein, lower triglyceride, lower sICAM, lower alanine aminotransferase, lower amylase, higher myeloperoxidase without higher CRP, higher catalase, higher nitric oxide, and higher radical scavenging	Lipid preparation of curcumin can lead to health-promoting effects in healthy middle-aged adults
Cox et al. (2020) [13]	Healthy older people aged 50-80 years (mean=68.1, SD=6.34)	Longvida®️ curcumin (400 mg daily containing 80 mg curcumin)	80 (40 curcumin and 40 placebo)	12-week double-blind, placebo-controlled, and partial replication study	Curcumin was associated with statistically significant results in cognitive performance and serum levels as below: improved WM at 12 weeks (Serial Threes and Sevens, and virtual Morris Water Maze), lower fatigue at four and 12 weeks (Profile of Mood States), lower mood-related confusion at four weeks, and higher glucose	Longvida®️ preparations of curcumin improve WM and mood in healthy older adults. The improvements in hippocampal functional domains hold promise for alleviating cognitive decline in similar populations
Rainey-Smith et al. (2016) [14]	Healthy older adults 40-90 years old with no cerebrovascular disease or cognitive impairment	1500 mg/day Biocurcuma™️	96 (160 initially enrolled)	12-month randomized, placebo-controlled, and double-blind study	Curcumin was associated with statistically significant results in time to treatment for the MoCA (P<0.050). At six months, cognitive function declined in the placebo group, but not in the curcumin group. No other differences were detected	The study suggests curcumin can potentially prevent cognitive decline, but more research is needed to investigate changes in cognitive measures in conjunction with serum biomarkers
Small et al. (2018) [15]	Non-demented adults with normal brain aging or mild cognitive impairment age 50-90 years old	Bioavailable form of curcumin (Theracurmin®️ containing 90 mg of curcumin twice daily)	40 (21 curcumin and 19 placebo)	18-month randomized, double-blind, and placebo-controlled trial	Curcumin in the form of Theracurmin®️ was associated with statistically significant results in improved verbal memory (p=0.002), improved visual memory (p=0.006), improved attention (p<0.0001), and decreased FDDNP binding in the amygdala (p=0.04) and hypothalamus (p=0.02) as observed on PET	The results suggest daily curcumin in the form of Theracurmin®️ can improve memory and attention in non-demented adults. The coinciding FDDNP-PET results correlate these functional benefits with decreases in the neurodegenerative proteins amyloid and tau in brain areas controlling emotions and recall
Kuszewski et al. (2020) [16]	Overweight or obese (BMI 25-40 kg/m^2^) middle-aged and older adults (50-80 years)	Fish oil (2000 mg/d docosahexaenoic acid (DHA)+400 mg/d eicosapentaenoic acid (EPA), curcumin (160 mg/d), and combination	152 (134 completed)	16-week double-blind, and placebo-controlled intervention trial	⁠The following statistically significant findings were observed: isolated curcumin caused improvement in CVR in a WM test and improved performance in a verbal memory test for males only, isolated fish oil caused improved CVR for processing speed tests in males only, and combining fish oil with curcumin did not add benefits	Improvements in processing speed following fish-oil consumption and memory following curcumin consumption in 50-80 years old males may correlate with improved circulatory function or CVR. Sex differences need more investigation
Lee et al. (2014) [17]	Pre-diabetic adults (BMI 18.5-30 kg/m^2 ^and fasting glucose 100-125 mg/dL), age 60 and older	Turmeric (1 g), cinnamon (2 g), or both (1 g turmeric+2 g cinnamon)	48	Double-blind metabolic study	Curcumin in the form of dietary turmeric was associated with statistically significant results compared to cinnamon as follows: WM increased from 2.6 to 2.9 out of 3.0 (p=0.05); WM was inversely related to insulin resistance (R of 34.5% and p<0.01), not with serum biomarkers. WM responses to turmeric had significant beta-coefficients for turmeric, BMI, and insulin/glucose area under the curve	Consumption of turmeric with white bread improves WM regardless of body fat, glycemia, insulin, or biomarkers. Findings suggest turmeric may benefit the cognitive performance of adults with pre-diabetes
Thota et al. (2020) [18]	Adults with high risk of type 2 diabetes mellitus age 30-70 years	Curcumin (180 mg/day)	29 (15 placebo and 14 curcumin)	12-week randomized, double-blind, and placebo-controlled study	Curcumin is associated with statistically significant reductions in the following serum biomarker levels: GSK-3β (−2.4±0.4 ng/mL, p=0.00001) as compared to placebo (p=0.0068 ), IAPP (−2.0±0.7 ng/mL, p=0.01) as compared to placebo (p=0.0163), and HOMA2-IR (p=0.0142) compared to placebo (p=0.9747)	Curcumin significantly reduces serum levels of insulin resistance-biomarkers GSK-3β and IAPP, suggesting curcumin can decrease risk of type 2 diabetes and Alzheimer’s dementia
Das et al. (2023) [19]	Cognitively impaired adults aged 55-75 years with MMSE scores (14-24, all values inclusive)	CGM (400 mg x 2/day), USC complex with 95% purity, and placebo	48 (16 per group)	Six-month randomized, double-blinded, placebo-controlled, and 3-arm, 3-sequence comparative study	Curcumin in the form of CGM compared to placebo and USC was associated with statistically significant results as follows: improved MMSE scores, improved Geriatric Locomotive Function Scale Scores, and improved serum biomarkers (brain-derived neurotrophic factor, beta-amyloid 42, tau protein, Interleukin 6, and tumor necrosis factor-α)	Curcumin in the form of CGM significantly hindered the development of Alzheimer’s disease, as observed in improved cognitive and locomotive functions as well as serum biomarkers. The bioavailable form of CGM is more effective than USC and the placebo
Ringman et al. (2012) [20]	Adults aged greater than 49 years with mild to moderate as well as probable, Alzheimer’s disease (MMSE scores 17-29), have a mean age of 73.5 years and mean MMSE score of 22.5	Curcumin C3 Complex®️ (2 g/day or 4 g/day)	36	24-week randomized, double-blind, and placebo-controlled study with a 24-week open-label extension	Curcumin as Curcumin C3 Complex®️ was associated with no difference in clinical measures or serum biomarker measures compared to placebo [20]. ⁠Serum levels of curcumin were low (7.32 ng/mL), and there were insignificantly lower serum hematocrit and increased serum glucose associated with curcumin [20]. One subject (8%) in the placebo group withdrew due to worse memory, and five of twenty-four subjects (21%) withdrew in the curcumin group due to gastrointestinal (GI) side effects	Curcumin was well-tolerated despite the GI side effects [20]. No clinical or biochemical efficacy of Curcumin C3 Complex®️ was found in Alzheimer's disease [20]. The lack of efficacy may be attributed to the limited bioavailability of the compound as suggested by preliminary data
Alimadadi et al. (2022) [21]	Major depressive disorder patients aged 18-55 years with normal literacy	Curcumin (500 mg twice daily) plus sertraline vs. placebo plus sertraline	120 (60 curcumin and 60 control)	12-week double-blind and randomized clinical trial	Curcumin compared to placebo was associated with statistically significant (P<0.001) results as follows: cognitive improvement in forwarding and backward digit span (η²=0.286 and η²=0.390), Trial Making Test-A and B (η²=0.161 and η²=0.026), verbal fluency task: letter and category (η²=0.105 and η²=0.128), Tower of London (η²=0.211), and inflammatory markers: interleukin 6 and 1β (η²=0.121 and η²=0.097)	Curcumin significantly enhanced cognitive performance and attenuated serum inflammatory markers for major depressive disorder patients, suggesting curcumin is a potential adjuvant treatment for depression
Putri Laksmidewi et al. (2024) [22]	Cooperative patients with cervical cancer treated with carboplatin-pacitaxel chemotherapy at risk of CICI; treatment group mean age 46.74 (SD: 10.67) and placebo mean age 50.46 (SD: 7.02)	Curcumin extract (240 to 400 mg intermittently)	78 (39 curcumin and 39 placebo)	24-week double-blind, randomized, and placebo-controlled trial	Curcumin compared to placebo was associated with statistically ⁠significant improvements in cognitive function as follows: Stroop test (∆ median 8.57 vs. 2.46; Z −4.503 vs. −1.762; p<0.0001 vs. 0.078) and MoCA-Ina (∆ mean 1.53 vs. 0.72; Z −2.99 vs. −2.05; p<0.003 vs. 0.04) [22]. ⁠No difference between groups was observed for drop-out, mortality, or adverse drug response	Curcumin extract administered intermittently with dose escalation significantly improved cognitive function in patients with CICI as compared to placebo, and it has a good safety profile
Ng et al. (2022) [23]	Community-dwelling adults in Singapore age 55 years and older (mean age 65.9 and SD 7.4)	Curcumin-rich curry consumption (frequency: never/rarely, occasionally, often, and very often)	2751	4.5-year longitudinal observational study	Curcumin in the form of curry consumption was associated with statistically significant and higher scores on the following neurocognitive tests as listed: Digit Span-Backward with higher scores in the "very often" and "often" groups, Verbal Fluency (Animals) with higher scores in the "very often" group, and Block Design with higher scores in the "occasional," "often,” and "very often" groups. Cohen’s d-effect size ranged from 0.130 to 0.186 for the significant differences observed.	Consumption of dietary curcumin was significantly associated with higher attention, short-term WM, visual-spatial constructional ability, language, and executive function among older adults in the community of Singapore. The findings suggest curcumin may directly and indirectly offer neuroprotective benefits, through metabolic, antiplatelet, and cardioprotective mechanisms

Results

Search Results

**Figure 1:**
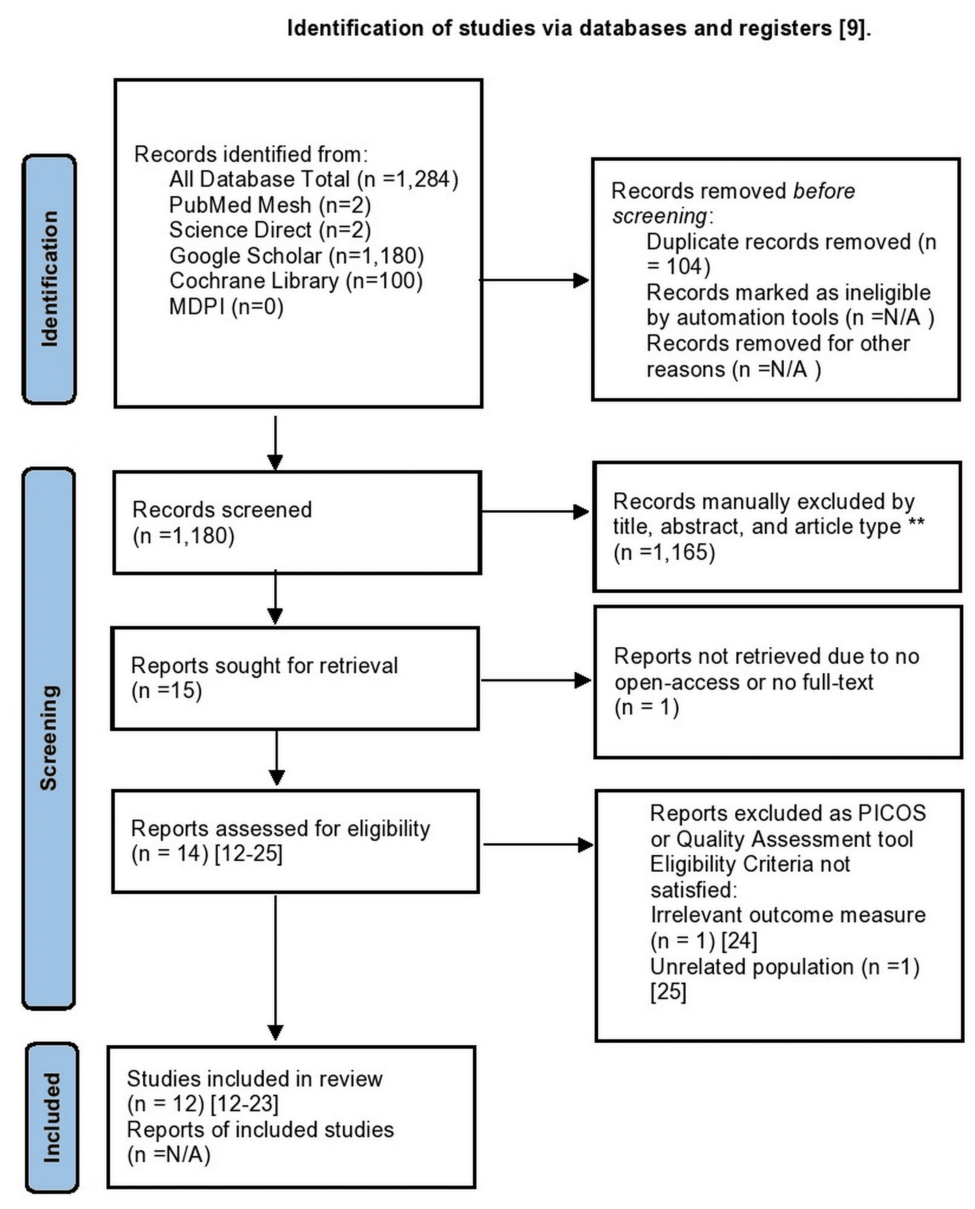
PRISMA flow diagram summary of search results ^**^Excluded article types are those that do not include open-access full-text articles, clinical trials, RCTs, cohort studies, human studies, or studies published in the last twenty years. MDPI: Multidisciplinary Digital Publishing Institute; PRISMA: Preferred Reporting Items for Systematic Reviews and Meta-Analyses; PICOS: Population, Intervention, Comparator, Outcome, Study Design; RCT: randomized controlled trial

The search strategy identified 1284 studies across five databases, including PubMed, Science Direct, Google Scholar, Cochrane Library, and MDPI. After filtering out 104 duplicate articles, AJF and AP screened the remaining 1180 studies for PICOS criteria by scanning each study's title, abstract, and design type. Ultimately, 15 full-text articles were retrieved. Subsequently, we excluded one clinical trial because no open-access or full-text version was available [7]. The remaining 14 studies were evaluated for internal validity using quality assessment tools. Two of the 14 full-text or open-access studies were excluded due to irrelevant outcomes and population measures and are not included in the tables [24,25]. Ultimately, 12 studies met appropriate internal validity standards for inclusion in this systematic review, as seen in Table 2 and Table 3 [12-23]. Figure 1 shows the findings of the study selection process [9].

Internal Validity of Each Study

Each RCT was thoroughly assessed for bias using the RoB2 assessment tool, receiving a rating of "Low," "High," or "Some Concerns". The included studies were of high quality and low bias. Table 2 presents the RoB2 quality assessment tool for screening each RCT, demonstrating the results of our evaluation process [10].

The single retrospective cohort study was thoroughly assessed for bias using the Newcastle-Ottawa tool. The included study was of high quality and low bias, as seen by the high number of stars awarded. Table 3 displays the Newcastle-Ottawa quality assessment results for the included cohort study [11].

Study Characteristics of Each Study

Among the RCTs, four found significant efficacy in healthy middle-aged and older adults, three found significant efficacy in adults with obesity and pre-diabetes, two found efficacy in adults with pre-existing cognitive impairment and dementia, one found significant efficacy in adults with major depressive disorder (MDD), and one found efficacy in adults with chemotherapy-induced cognitive impairment (CICI) [12-22]. The single included retrospective cohort study also found significant dose-specific improvements in cognitive performance with bioavailable curcumin, suggesting that the therapeutic effects of curcumin are dose-dependent and increase with the frequency of curcumin consumption [23]. We detailed the characteristics of each study in Table 4.

Discussion

Comparative Analysis of Key Findings Between Studies

In total, 10 of the 11 RCTs demonstrated statistically significant improvements in outcome measures for treatment groups administered bioavailable curcumin compared to alternate supplementation, as outlined in Table 4 [12-19,21,22]. Curcumin significantly improved, p <0.05, working memory and mood in healthy older adults with no prior cognitive or psychosocial impairments, with a 20% improvement in cognitive performance, suggesting potential benefits in preventing cognitive decline in healthy non-demented adults [12-15]. Curcumin significantly improved, p <0.01, cognitive performance, verbal memory, and cerebrovascular reactivity, as reflected in changes in blood flow to the brain, which can be measured with various imaging techniques in adults with obesity and prediabetes, indicating benefits for metabolic and cognitive health [16-18]. Curcumin showed significant improvements, p <0.05, in cognitive and locomotive function as well as a reduction in biomarkers as listed in Table 4, with improved MMSE scores by 15% for adults with pre-existing cognitive impairment and dementia, highlighting its potential for palliative neuroprotection [19,20]. Curcumin significantly enhanced, p <0.05, cognitive performance scores by 18% and attenuated serum inflammatory biomarkers in adults with major depressive disorder, suggesting curcumin as an adjuvant treatment for depression [21]. Curcumin significantly improved, p <0.05, cognitive function tests with a good safety profile in adults with CICI, supporting the use of curcumin in managing the cognitive side effects of chemotherapy [22]. One single RCT by Ringman et al. showed no statistically significant outcome improvement. However, the study should have used an appropriately bioavailable form of curcumin, stressing the importance of utilizing bioavailable pharmacologic formulations combined with piperine or lipid compounds [20,6]. This systematic review found that in 11 of the 12 included studies, cognitive performance and serum biomarkers were significantly improved in groups that consumed bioavailable forms of curcumin compared to placebo or alternate interventions [12-19,21-23]. All-together, results found that there is a statistically significant improvement in cognitive performance and serum biomarkers for adults age 18 and older with the following characteristics: non-demented healthy adults, adults at risk of or already diagnosed with metabolic disorders, adults with pre-existing cognitive decline, adults with mood disorders, and adults with cognitive chemotherapy side effects. These significant findings were consistent across multiple patient populations and study types, suggesting that bioavailable forms of curcumin can effectively improve cognitive performance and serum biomarkers across various neurocognitive disorders [12-23].

Implications of Results in Clinical Practice

Our comprehensive systematic review delves into the potential of curcumin to significantly impact cognitive performance and serum biomarkers in adults at risk of or already experiencing various degrees of neurocognitive disorders. By synthesizing data from 12 studies, we aimed to gain deeper insights into curcumin's potential as a preventive and palliative treatment for the cognitive health of adults aged 18 and above. This review involved a meticulous analysis of the methods and outcomes of 11 clinical trials and one cohort study, explicitly determining whether curcumin significantly enhances cognitive performance or serum biomarkers in adults at risk of or already diagnosed with cognitive disorders. Our main objective was to gather compelling evidence that advocates for integrating curcumin into standard therapy to enhance neurocognitive health, and we aim to guide clinical decisions regarding the preventive and therapeutic use of curcumin for neurocognitive disorders. This research offers a promising outlook for neurocognitive health, expanding potential treatment options by exploring curcumin [12-23].

Neurocognitive disorders encompass a wide range of conditions, with Alzheimer's disease being the most well-understood and extensively studied. The disease, characterized by inflammation and neuronal damage, leads to the accumulation of beta-amyloid proteins and a tau cascade, ultimately resulting in the breakdown of the cortical white matter of the brain, visible on imaging. Functional impairments eventually arise, impacting activities of daily living differently, depending on the rate of progression of cognitive decline [26]. The degree of neurocognitive decline is assessed by implementing cognitive performance tests, brain images, and serum biomarkers [4,26]. Symptoms of Alzheimer's and other neurodegenerative diseases progress gradually with age and include a decline in cognitive performance exams like the MMSE and the MoCA [2,3]. 

Current therapeutic interventions for neurocognitive disorders include cholinesterase inhibitors and N-methyl-D-aspartate receptor antagonists, such as donepezil and memantine. However, these treatments do not reverse or cure actively progressing neurocognitive disorders but focus on preventing further decline. Modifiable risk factors for Alzheimer's prevention include diabetes, obesity, social isolation, sedentary lifestyle, alcohol use, and hypertension. Recent treatment strategies emphasize the rapid detection of serum biomarkers to identify and delay early cognitive decline. New pharmacologic strategies aim to combat inflammatory biomarkers, including beta-amyloid, tau, and general inflammation [26]. Curcumin, a polyphenol of turmeric, has been shown to have antioxidant and anti-inflammatory properties, making it a promising treatment approach against neurodegeneration [6]. This systematic review explores the potential of curcumin to inhibit the development of inflammatory serum biomarkers and improve cognitive health.

Curcumin's main medicinal benefits stem from its antioxidant and anti-inflammatory properties, which make it a promising treatment approach against neurodegeneration. Moreover, curcumin offers several advantages, including a high safety profile, low toxicity, easy accessibility, and low cost. It also has minimal drug-to-drug interaction; however, curcumin's limitations include gastrointestinal issues and skin color changes at extreme doses. Another notable drawback of curcumin therapy is its limited bioavailability, primarily due to inadequate absorption and swift excretion. However, this limitation can be overcome with enhancers like piperine in black pepper and liposome bodies, as they increase absorption through the gastrointestinal tract and blood-brain barrier [6,7]. This reassurance of safety and accessibility should instill confidence in curcumin's potential use and application.

The studies in this systematic review collectively suggest that bioavailable forms of curcumin are a promising treatment option for cognitive decline in adults 18 and older, as observed in cases both with and without neurocognitive disorders. Four of the included clinical trials, with 318 patients enrolled and 254 patients completing the trials, found that curcumin was associated with statistically significant improvements in cognitive performance and serum biomarkers in healthy, non-demented adults [12-15]. Three of the included RCTs, with 229 patients enrolled and 211 patients completing the trials, found curcumin was associated with statistically significant improvements in cognitive performance and serum biomarkers in adults at risk of type 2 diabetes or other metabolic disorders who were either overweight, obese, metabolically unstable as apparent on labs, or actively pre-diabetic [16-18]. One clinical trial, with 48 patients enrolled and completing trials, discovered that a highly absorbable type of curcumin was linked to a statistically significant enhancement in cognitive function and serum biomarkers among adults experiencing pre-existing cognitive impairment or dementia [19]. One of the clinical trials, with 120 patients enrolled and completing the study, discovered that curcumin was linked to a statistically significant enhancement in cognitive function and serum biomarkers in adults with major depressive disorder [21]. One RCT, with 78 patients enrolled and completing the study, found that curcumin was associated with a statistically significant improvement in cognitive performance for adults experiencing CICI [22]. The single cohort study, with 2751 patients enrolled and completing the study, found that a higher frequency of curcumin consumption in the form of dietary curry among Singaporean community dwellers was associated with a statistically significant improvement in attention, short-term working memory, visual-spatial constructional ability, language, and executive function among older adults [23]. Diverging from the majority of the trials, only one of the clinical trials found no statistically significant effects of curcumin in adults with likely dementia; however, this is due to the low bioavailability of the curcumin formulation for this single case [20]. Ultimately, 11 of the 12 studies (nearly 92%) found that curcumin was associated with statistically significant improvement in cognitive performance or serum biomarkers for each patient population analyzed [12-19,21-23]. Collectively, the studies suggest that curcumin has significant neuroprotective effects and can significantly prevent cognitive decline or neurodegeneration in non-demented healthy adults, adults at risk of or already diagnosed with metabolic disorders, adults with pre-existing cognitive decline, adults with mood disorders, and adults with cognitive chemotherapy side effects [12-23]. The beneficial results are not acute and are more effective after several weeks of treatment [13]. Thus, similar to the findings of a prior study by Tsai et al., this review also indicates that the statistically significant benefits of curcumin are dose-specific and affect each cognitive domain differently depending on the administration regimen, characteristics of the patient population, pharmacologic form of curcumin, and length of treatment. While not all cognitive domains and their corresponding brain regions responded uniformly, curcumin consistently and significantly benefited the working memory domain of cognitive performance in all the studies [8,12-23]. This potential of curcumin to prevent cognitive decline expands the options for promoting cognitive health.

Study limitations

There are several limitations to this study. First, the wide range of neurocognitive disorders assessed in this review, each with a different baseline pathophysiology, may have contributed to heterogeneous responses to curcumin, thus affecting the uniformity of the data. Second, this study did not include a meta-analysis, preventing the determination of how differences in neurocognitive disorders might have influenced the results. In future studies, conducting a meta-analysis of the data would be beneficial. Third, the number of available trials was limited. However, the data remains significant enough to suggest that curcumin consistently benefits patients with various neurocognitive disorders, particularly in the working memory domain. Despite the limited sample size, the outcome was objectively consistent and statistically significant across the studies included. Finally, most studies employed per-protocol analysis, excluding data from non-compliant participants. Including participant data using an intention-to-treat analysis would reduce bias and provide a more accurate representation of the outcomes. Future clinical trials should follow, using the intention-to-treat analysis, as primarily recommended by Tsai et al. [8].

## Conclusions

This systematic review found that curcumin supplementation causes a statistically significant improvement in cognitive performance as well as serum biomarkers for adults age 18 and older with the following characteristics: non-demented healthy adults, adults at risk of or experiencing metabolic disorders, adults with pre-existing cognitive decline, adults with mood disorders, and adults with cognitive chemotherapy side effects. The studies indicate that bioavailable curcumin offers therapeutic benefits across different stages and causes of cognitive decline, improving working memory and preventing neurodegeneration in a dose-specific manner that remains to be specified. This evidence supports the use of curcumin as a preventive, adjunctive, or alternative therapy for neurocognitive disorders. However, curcumin has some gastrointestinal side effects to consider during shared medical decision-making with a patient before treatment. Future meta-analyses and RCTs should follow and implement the intention-to-treat outcome analysis.
